# Cutaneous squamous cell carcinoma of the scalp surrounding a ventriculoperitoneal shunt in a renal transplant recipient with spina bifida

**DOI:** 10.1016/j.jdcr.2024.12.026

**Published:** 2025-01-09

**Authors:** Po-Han Ho, Jessica Saoub, Farah Abou-Taleb, Nancy Fischbein, Curtis Chong, Anne Lynn S. Chang

**Affiliations:** a Department of Dermatology, Stanford University School of Medicine, Redwood City, California; b Department of Radiology, Stanford University School of Medicine, Stanford, California; c Department of Medicine-Oncology, Stanford University School of Medicine, Redwood City, California

**Keywords:** cutaneous oncology, cutaneous squamous cell carcinoma, immunosuppression, locally advanced disease, multidisciplinary care, transplant, ventriculoperitoneal shunt

## Introduction

The incidence of nonmelanoma skin cancers of the head and neck region is projected to continue increasing over the next decades^1^, affecting both immunocompetent and immunosuppressed patients. Cutaneous squamous cell carcinoma (CSCC) of the scalp can present significant management challenges in immunosuppressed patients, with added complexity when a subcutaneous device such as a ventriculoperitoneal (VP), ventriculoatrial or ventriculopleural shunt catheter is near the tumor. Ventricular shunts drain excess cerebrospinal fluid from the ventricles, traverse the skull, and tunnels under the scalp and neck skin before draining into the peritoneal, right atrial, or pleural space. Since the scalp is an often photo-exposed site, cutaneous malignancies can occur on or near such shunt catheters, and recognition of this possibility is vital to safe diagnosis, effective treatment, and avoidance of potentially life-threatening infections.

At present, we find only a single case report in the literature regarding the management of nonmelanoma skin cancer and subcutaneous shunts, namely a recurrent neck basal cell carcinoma treated with excision and shunt removal^2^ (Table I) With no previous reports of CSCC involving VP shunts (VPS), we present a unique case of an immunosuppressed patient with an aggressive scalp CSCC involving her VPS with extensive calvarial and dural involvement leading to inoperability.

**Table I:** Cases in the literature of nonmelanoma skin cancers (NMSCs) involving *either* a ventriculoperitoneal shunt (VPS) (top portion of table) *or* cutaneous squamous cell carcinoma (CSCC) with calvarial involvement (middle portion of table)

Primary nonmelanoma skin cancer surrounding VPS						
Primary NMSC tumor type	Reason for VP shunt placement	Immunosuppression (type)	Calvarial/dural involvement	Treatment after discovery of VP shunt involvement	Outcome	Ref.
BCC∗	Spina bifida	Renal transplant with immuno-suppression (tacrolimus, mycophenolate mofetil, prednisone)	Local recurrence on neck skin abutting VP shunt; calvarial/dural involvement not mentioned	Wide excision with a peripheral margin of 1 cm, removal of the involved shunt tract, and intraoperative frozen sections assessment	Clinically disease-free at 24 months after last recurrence	2 (no PMID)

Scalp CSCC with calvarial invasion (without VPS)						
Tumor type	Immuno-suppression (type)	Tumor location (longest reported diameter, other risk factors)	Calvarial/dural involvement	Treatment	Outcome since CSCC diagnosis	Ref.
CSCC	No	Parieto-occipital scalp (5 cm)	Yes/Yes	Wide excision with vault prosthetic cover, scalp transposition flap, and postoperative adjuvant radiotherapy	Tumor clearance: skull bone excised with free margins; well-healed scalp skin defect at 2 months postsurgery	PMID 36938224
CSCC	No	Parieto-occipital scalp (6 cm, history of childhood injury to site)	Yes/Yes	Total tumor resection, repair of dura, and repair of skin defect by advancement flap	Skin well healed at 3 months follow-up postsurgery with no evidence of metastasis	PMID 36268452
CSCC	No	Parietal scalp (8.5 cm, distant history of synthetic hair implants at same site as CSCC)	Yes/No macroscopic dural invasion	Radiotherapy followed by tumor resection and reconstruction with synthetic bone material, free latissimus dorsi muscle flap, and skin graft	No recurrence or metastasis at 9-month postsurgery follow-up	PMID 35923983
CSCC	No	Occipito-temporo-parietal scalp (9 cm, perivascular invasion, tumor emboli)	Yes/No	Tumor resection, deep curettage of the cranial bone involved by the tumor, and wound closure with several rotation advancement flaps	Not reported	PMID 35620944
CSCC	No	Fronto-parietal scalp (size not reported, poorly differentiated, recurrent)	Yes/Yes	Patient decided not to pursue treatment	>12 months survival after last recurrence	PMID 35119380
CSCC∗	No	Frontal scalp (15 cm, no other high-risk features reported)	Yes/Yes	Excision with 1 cm margin, cutaneous reconstruction with a scalp graft and free thigh flap transfer, postoperative radiotherapy	No evidence of recurrence at 24 months follow-up postsurgery	PMID 33194281
CSCC	No	Parietal scalp (size not reported, poorly differentiated)	Yes/No	Mohs surgery, ablative erbium laser to outer table of the calvarium	<12 months survival after surgery/laser	PMID 28736885
CSCC	No	Frontal scalp (20 cm, prior burn site)	Yes/Yes	Resection of tumor and calvarial bone	13 months of survival after initial resection followed recurrence	PMID 16127670
CSCC	Yes (HIV)	Parietal scalp	Yes/Unknown	None	<2 months survival after diagnosis of calvarial involvement	PMID 15765601

Primary nonmelanoma skin cancer surrounding VPS and invading calvarium						
Primary NMSC tumor type	Reason for VP shunt placement	Immunosuppression (type)	Calvarial/dural involvement	Treatment after discovery of VP shunt involvement	Outcome	Ref.
CSCC	Spina bifida	Renal transplant with immunosuppression (tacrolimus, mycophenolate mofetil)	Local recurrence on parietal scalp over VPS with calvarial/dural involvement	Inoperable due to dural involvement; nonresponsive to pembrolizumab, tumor progression under cetuximab; currently on carboplatin/paclitaxel	CSCC still present, >14-month follow-up from diagnosis of calvarial invasion	Current case

Our case combines *both* VPS involvement and calvarial involvement. Only one NMSC involving a VPS has been previously reported, consisting of a BCC,^2^ shown below, but it did not involve calvarium. CSCC abutting a VPS has not been previously reported. The BCC case shares several similarities with our present CSCC case: both patients had spina bifida and were renal transplant patients on immunosuppression. BCCs are generally less aggressive than CSCC, and the BCC was excised with disease-free outcome at 24 months. In comparison, our CSCC case was deemed inoperable, and multiple lines of systemic treatment were needed. In both cases, the NMSCs were around the VPS, without evidence of tumor invading or tracking *within* the VPS. The middle portion of the table shows examples of CSCC cases with calvarial involvement (without shunts) and describes immunosuppression status, treatments, and outcomes.

*BCC*, Basal cell carcinoma; *CSCC*, cutaneous squamous cell carcinoma; *NMSC*, nonmelanoma skin cancer; *PMID*, PubMed reference number; *VPS*, ventriculoperitoneal shunt.

∗ The cases with the longest reported survival outcomes are indicated with an asterisk, though cases cannot be directly compared.

## Case report

A woman in her 50s with spina bifida and a left-sided VPS (placed as a newborn) presented to dermatology clinic with a 4-year history of an enlarging mass on the left scalp, which was obscured under thickly matted hair. Twenty years ago, end-stage renal disease from spina bifida-related obstructive uropathy led to a renal transplant, managed on mycophenolate mofetil and tacrolimus for the past 10 years.

**Fig 1:**
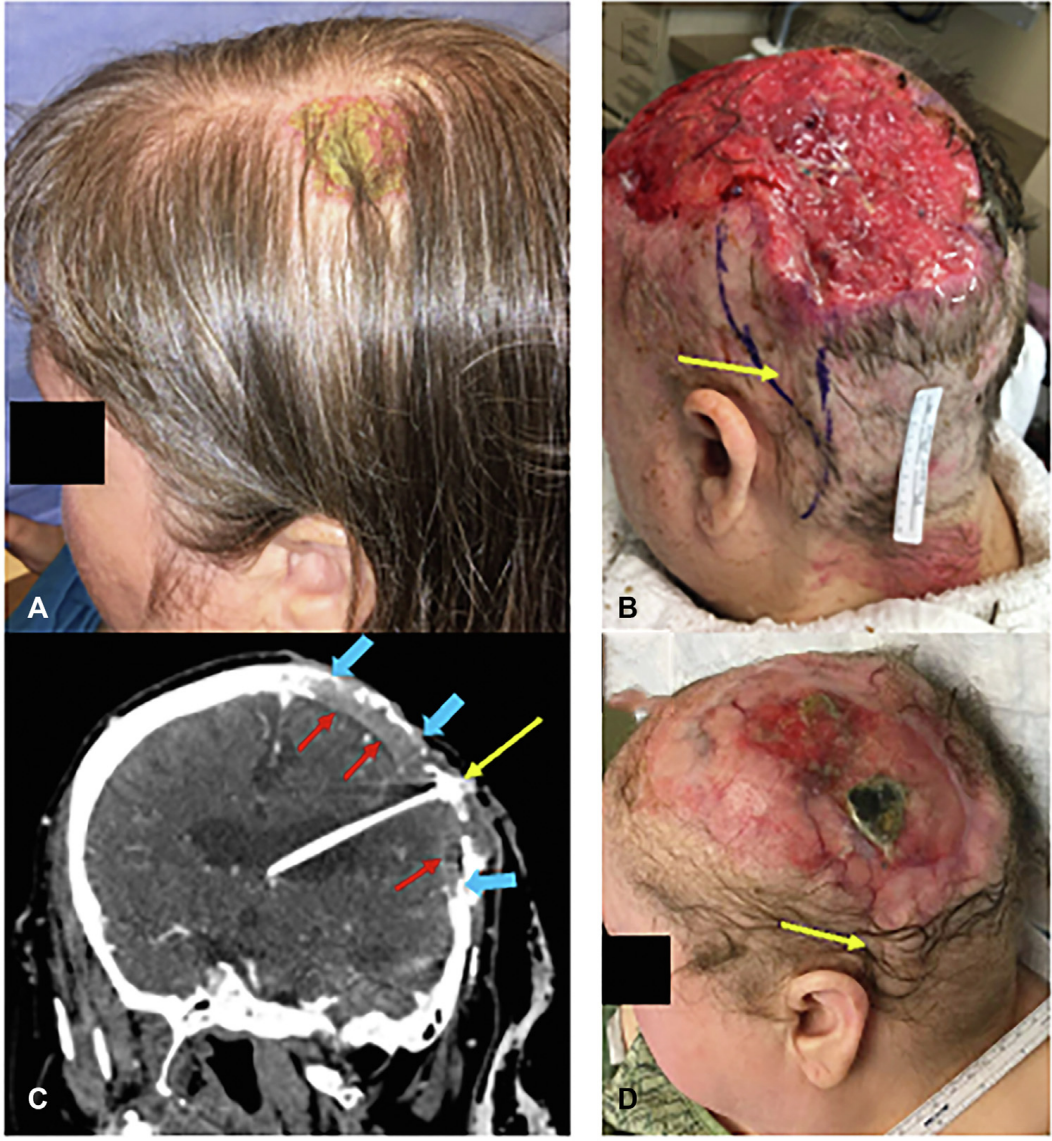
Cutaneous squamous cell carcinoma (CSCC) of the scalp in a spina bifida patient with a ventriculoperitoneal shunt (VPS) and a renal transplant on immunosuppression. (**A**) Photograph taken immediately prior to left scalp biopsy, which showed CSCC *in situ* (CSCCIS*)*, taken 6 years prior to (**B**), before any treatment. The CSCCIS was treated with 5-fluorouracil (5-FU) cream, but development into CSCC necessitated Mohs surgery for the invasive component, with the *in situ* component treated with additional 5-FU cream. (**B**) The patient did not follow up for several years and then presented with a 13 cm ulcerated plaque on the left scalp overlying the prior CSCC site. The outline of the retroauricular portion of the subcutaneous VPS catheter was visible and palpable (*yellow arrow*), extending from the ulcer to the neck. Biopsy at least 3 cm away from the shunt (white dashed circle) confirmed invasive CSCC with perineural invasion. (**C**) Computed tomography (CT) scan of the head showed the VPS catheter (*yellow arrow*) surrounded by extensive calvarial destruction (*blue arrows*) and dural involvement (*red arrows*) by the CSCC. The neurosurgery team determined the CSCC was inoperable due to the extent of osseous and dural involvement. (**D**) The patient underwent multiple lines of systemic therapy over the next 13 months, with CSCC response evident (*yellow arrow*) after carboplatin/paclitaxel.

Six years ago, she had a biopsy-proven CSCC *in situ* (CSCCIS) on the left scalp (Fig 1, *A*, photograph taken immediately prior to this CSCCIS biopsy). It was treated with 5-fluorouracil (5-FU) 5% cream twice daily for >2 weeks with discontinuation from irritation and visible shrinkage. Seven months later, regrowth and repeat biopsy indicated invasive CSCC, and Mohs surgery removed the invasive CSCC component. 5-FU cream was recommended again for the surrounding *in situ* disease. Due in part to the COVID-19 pandemic, the patient did not seek dermatologic care for the following 4 years.

Upon presentation, she had no neurologic deficits nor systemic infection symptoms, and debridement of the matted hair revealed a 13 × 9 cm ulcerative plaque. Significantly, the outline of her VPS was visible within the ulceration and palpable under the intact scalp skin, without obvious shunt exposure (Fig 1, *B*). After confirmation of shunt location with a historical head computed tomography (CT) scan in the medical record, the patient consented to a skin biopsy on the ulcer’s periphery, >3 cm from the shunt, which revealed poorly differentiated CSCC with perineural invasion.

Subsequent CT imaging showed extensive calvarial and dural involvement by the CSCC around the VP catheter insertion site (Fig 1, *C*). No nodal or distant metastases were present, confirming locally advanced CSCC (American Joint Commission on Cancer seventh edition Stage T4*N*0M0). Neurosurgical consultation deemed surgical resection infeasible due to disease extent. Although the VPS was not functioning correctly, it could not be easily removed. Meropenem was initiated for infection prevention, in addition to her chronic sulfamethoxazole/trimethoprim usage due to immunosuppression.

Given the life-threatening nature of the CSCC and stable renal function, the patient and her nephrologist opted to discontinue mycophenolate mofetil and then tacrolimus. She was maintained on low-dose prednisone monotherapy, with good renal function and no evidence of transplant rejection. Over the next 13 months, the CSCC was managed by the medical oncologist with pembrolizumab (4 cycles) without response during treatment (though pseudo-progression and/or delayed treatment responses after discontinuation are possible). She switched to cetuximab (12 cycles) with disease progression prompting discontinuation and then carboplatin and paclitaxel, with visible disease improvement (Fig 1, *D*). She remains without neurologic deficits, continues chemotherapy, and seeks ongoing neurosurgery team input in case CSCC shrinkage leads to operability.

## Discussion

This case highlights several important learning points. First, obtaining a history of any subcutaneous device within the vicinity of anticipated biopsies or excisions is critical to avoid breaking the sterility of the device and risking infection (besides shunts, reservoirs may be present on the scalp). Second, if the device location is unclear on physical examination, correlation with imaging (CT, magnetic resonance imaging, or ultrasounds) in the medical record or real-time ultrasound may assist with avoiding procedures too close to the device. Third, if the neoplasm requiring biopsy or excision is near or abutting the device, immediate multidisciplinary consultation (eg, neurosurgery, otolaryngology, and/or plastic surgery teams) is important to plan the optimal procedural approach that minimizes infection risk.

In this case, early initiation of multidisciplinary consultation was urgent as the patient was a solid organ transplant recipient (SOTR) on immunosuppression from mycophenolate mofetil and tacrolimus. In addition to immunosuppression, these drugs may promote CSCC by impairing keratinocytes’ ability to repair DNA and undergo apoptosis.^3^ Hence, transitioning off these medications may enhance more than one antitumor mechanism.

SOTRs may experience more frequent and/or rapid transformation of CSCCIS into invasive disease due to incomplete response to topical treatments,^4^^,^^5^ though this has not been extensively studied. In a case series involving 5 SOTRs with CSCCIS treated with 5% 5-FU cream twice daily for 3 weeks, none achieved a complete response.^5^ While larger studies are needed, this limited data suggest that topical 5-FU usage cannot be considered a definitive treatment for CSCCIS in SOTRs. Instead, excision should be prioritized, with early involvement of a multidisciplinary surgical team so that curative excision can be accomplished.^6^

While VP shunts have been reported to serve as conduits for gastrointestinal cancers metastasizing to the skin,^7^, ^8^, ^9^ the reverse situation of a skin primary cancer using the calvarial defect around the VPS as a conduit into internal organs (such as the brain) has not been reported. Awareness of this possibility is particularly critical in immunosuppressed patients at high risk for poor outcomes with CSCC, and shunt systems in proximity to CSCC should be carefully palpated and scrutinized on imaging studies to assess for tumor presence along the shunt apparatus.

## Conflicts of interest

Dr Chang has been a consultant and clinical investigator for Merck. Drs Chong, Fischbein, Abou-Taleb, Saoub, and Ho have no conflicts of interest to declare.
